# Delayed Presentation of Severe Blunt Liver Trauma Following a 12-Foot Fall: A Case Report of a Grade 4 Hepatic Injury With a Concurrent Grade 1 Renal Injury

**DOI:** 10.7759/cureus.58179

**Published:** 2024-04-13

**Authors:** Muiz M Malik, Naveed U Khan, Sultan Alkuwaiti, Hafiz Muhammad Hamza, Ayaz A Awan

**Affiliations:** 1 School of Medicine, Foundation University School of Health Sciences, Islamabad, PAK; 2 Surgery, Federal Government Polyclinic Hospital Islamabad, Islamabad, PAK; 3 Surgery, Tawam Hospital, Al Ain, ARE

**Keywords:** hepatic packing, parenchymal injury, conservative management, fall from height, blunt liver trauma

## Abstract

The delayed presentation of a 15-year-old female with a complex Grade 4 liver injury and a concurrent Grade 1 renal injury sustained from a fall exemplifies the heightened vulnerability of adolescents to blunt hepatic trauma. Unlike typical presentations where symptoms like abdominal pain and internal bleeding appear immediately, this case emphasises the potential for delayed manifestation, posing unique challenges for diagnosis and management. This case, managed at a leading trauma centre, underscores the distinct challenges compared to adult cases due to adolescents' larger space available for the organ and immature livers. While presenting more management complexity than typical splenic injuries, prompt intervention with emergency laparotomy and hepatic packing proved crucial for the patient's successful outcome. This case emphasises the critical role of early identification, vigilant monitoring, and strict activity restrictions post-operatively for optimal adolescent liver trauma management and serves as a reminder of the spectrum of potential injuries, including bile duct and vascular damage alongside contusions and haematomas.

## Introduction

Given its size and comparatively stable position, the liver is vulnerable to damage from blunt abdominal trauma. In blunt abdominal trauma, the spleen and liver together are responsible for 75% of the damage [1]. About 80% of all cases of renal trauma are secondary to blunt trauma, which most frequently results from high-velocity incidents like car crashes, sports collisions, or falls from great heights [2]. Despite being the second predominantly wounded organ in abdominal injury, the liver is the main cause of fatality after abdominal trauma. Even at the best trauma centres, managing liver trauma is still more difficult than managing splenic injuries. A high proportion of liver injuries were previously managed surgically. However, data show that 67 out of 100 laparotomies performed for blunt abdominal injuries are not therapeutic, and around 86% of liver traumas had undergone cessation of haemorrhage by the time surgical intervention was conducted [3]. The right lobe is most frequently affected because of its larger size and closeness to the ribs. More than 85% of the liver segments 6, 7, and 8 sustain the greater part of the damage when compressed against the static ribs, spinal column, or posterior abdominal wall. The diaphragm may allow pressure on the right hemithorax to spread, causing a contusion on the right lobe of the liver's dome [4]. Compared to adults, children are more likely to suffer from liver injury because of factors like their larger stature, less developed livers, incomplete development, and more flexible ribs. When extremely high venous pressure is transmitted to distant body regions during impact, these factors raise the risk of liver injury in children [5]. Hepatic lobe tearing can result from deceleration injuries, which also frequently affect the hepatic veins and inferior vena cava. A complete lobe may be damaged consequently due to a steering column injury. Liver trauma can result in bile duct damage, hepatic vascular injury, punctures, contusions, and subcapsular or intrahepatic haematomas [6].

## Case presentation

A 15-year-old female was brought to the emergency room of the Polyclinic Hospital in Islamabad on February 18, 2024, with a history of a 12-foot fall accident 12 hours earlier. On examination, she was responsive, oriented, and tachypneic, with a pulse rate of 120/min and a BP of 75/55 mm Hg. Examination of the abdomen disclosed dispersed tenderness, whereas guarding or rigidity was absent in the whole abdomen. The neurological examination was normal, with a GCS of 15/15. Ultrasound revealed free fluid in the upper abdomen, Morrison’s pouch, pelvis, and between the gut loops. On a computed tomography (CT) scan of the chest and abdomen with and without contrast, segment IVb of the liver showed no enhancement with attenuation of the portal vein and hepatic artery branches within it. The area was poorly delineated from adjacent segments II, III, and IVa. This was suggestive of parenchymal disruption of less than 75% of the left lobe of the liver. Linearly hypodense, non-enhancing areas were seen within the cortex of the right kidney, extending to less than 1 cm in depth and perpendicular to the renal capsule, suggesting small lacerations. A final diagnosis of Grade 4 hepatic injury with concurrent Grade 1 renal injury was made. A laparotomy was performed in an emergency when a liver laceration measuring 7 x 2.5 x 1.5 cm on the posterior surface with active haemorrhage was noted. Hepatic packing helped in stopping bleeding. Afterwards, conservative treatment, which included intravenous omeprazole 40 mg, 10 mg ketorolac tromethamine, 1 g meropenem trihydrate, 400 mg moxifloxacin, and somatostatin 0.5 mg, was given. The patient was sent for magnetic resonance cholangiopancreatography. Hepatic parenchymal laceration with diffuse oedema of the dominant portion of the left hepatic lobe was noted. All the hepatic vessels were intact. The postoperative period went without incident. Following a further 14 days of hospitalisation, the patient was discharged in good health. The patient was advised not to do any load-bearing activity for at least three to six months.

Table 1 shows the results of initial laboratory investigations pertaining to liver function. The findings indicate elevated levels of liver enzymes, indicative of liver damage, alongside other parameters suggesting potential impairment in liver function. Total protein and albumin levels are slightly low, while globulin levels are slightly elevated. Prothrombin time (PT) was normal, indicating adequate blood clotting ability. The serum bilirubin total is elevated, suggesting potential liver dysfunction. Haemoglobin levels are within normal limits.

**Table 1:** Laboratory investigations ALT: alanine aminotransferase; AST: aspartate aminotransferase; PT: prothrombin time; INR.: international normalised ratio; Hb: haemoglobin; u/L: international units per litre; mg/dL: milligrams per decilitre; g/dL: gram per decilitre.

Test	Result	Reference range
ALT	200	5–55 u/L
AST	50	9–40 u/L
Total protein	5	6–8 g/dL
Albumin	2.7	3–5 g/dL
Globulin	2.3	2.5–3.5 g/dL
PT	13	12 seconds (control value)
INR	1.0	Up to 1.2
Serum bilirubin total	1.8	0.1–1.2 mg/dL
Hb	13.2	12–15 g/dL

**Figure 1:**
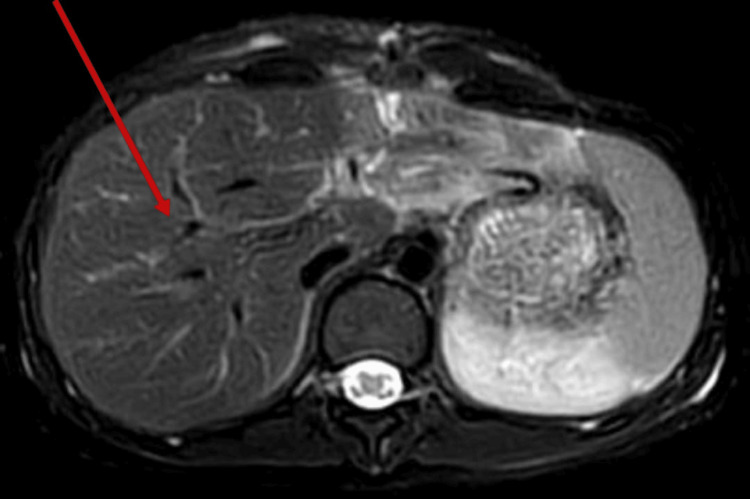
Magnetic resonance image showing a normal right liver lobe

**Figure 2:**
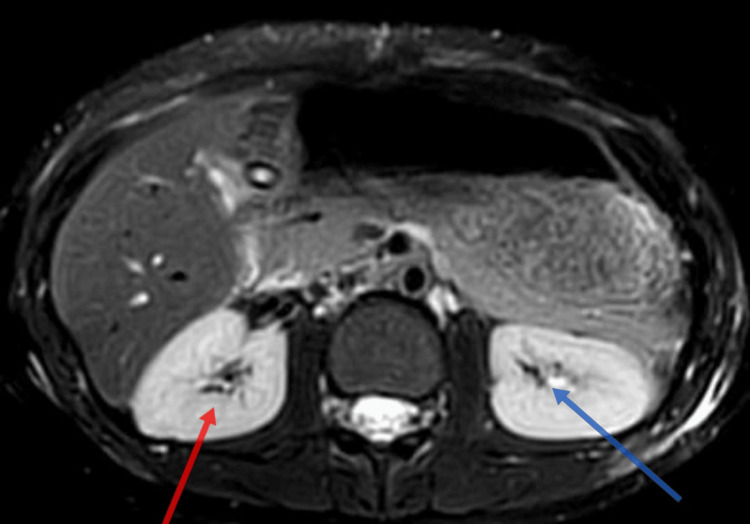
Magnetic resonance image showing right kidney laceration Red: right kidney; Blue: left kidney.

In Figure 1, a depiction of the normal right lobe of the liver after conservative management is shown. Subsequently, Figure 2 illustrates a right kidney laceration, highlighting a pathological condition necessitating further investigation and potential medical intervention.

**Figure 3:**
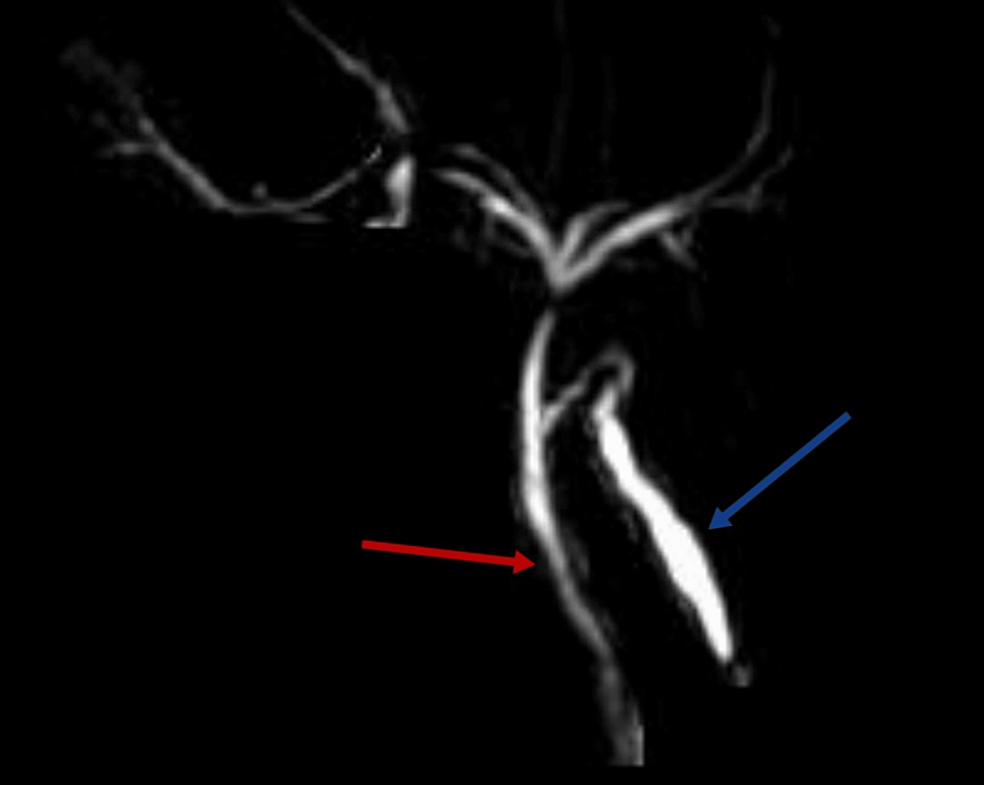
Magnetic resonance cholangiopancreatography showing normal gallbladder and common bile duct Blue: gallbladder; Red: common bile duct.

**Figure 4:**
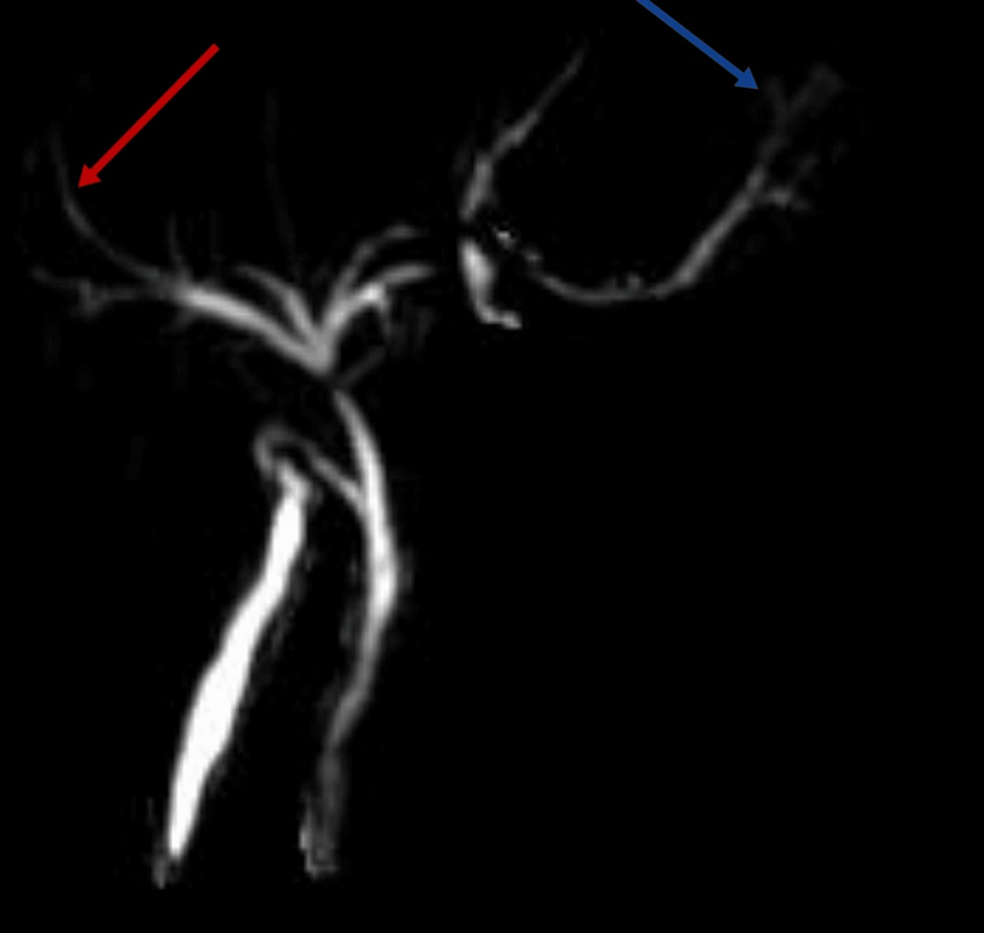
Magnetic resonance cholangiopancreatography showing the proximal end of the left hepatic duct with extravasation Red: right hepatic duct; Blue: left hepatic duct with extravasation.

**Figure 5:**
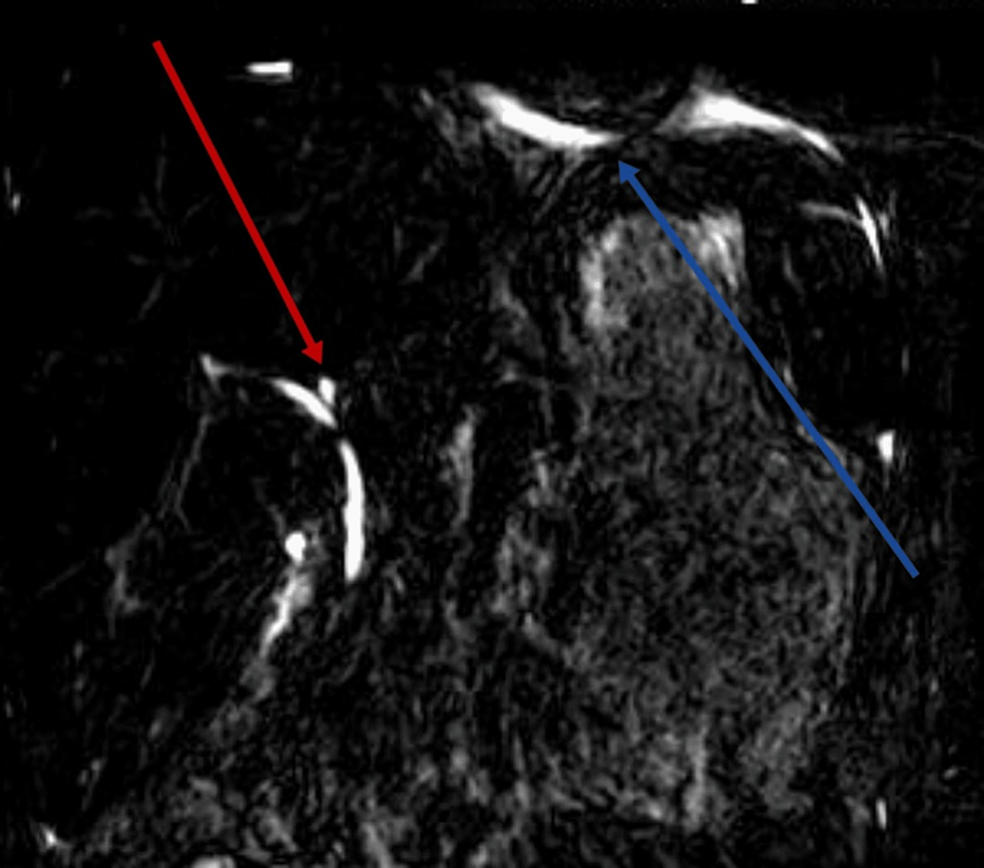
Magnetic resonance cholangiopancreatography showing bile spillage from the left hepatic duct Red: common hepatic duct; Blue: bile spillage from the left hepatic duct.

In Figures 3-5, magnetic resonance cholangiopancreatography (MRCP) provides detailed insights into the biliary anatomy. Figure 3 illustrates a normal gallbladder and common bile duct. Subsequent images (Figures 4, 5) delve into specific abnormalities, with Figure 4 revealing extravasation at the proximal end of the left hepatic duct, while Figure 5 showcases the spillage of bile from the left hepatic duct and the normal common hepatic duct.

**Figure 6:**
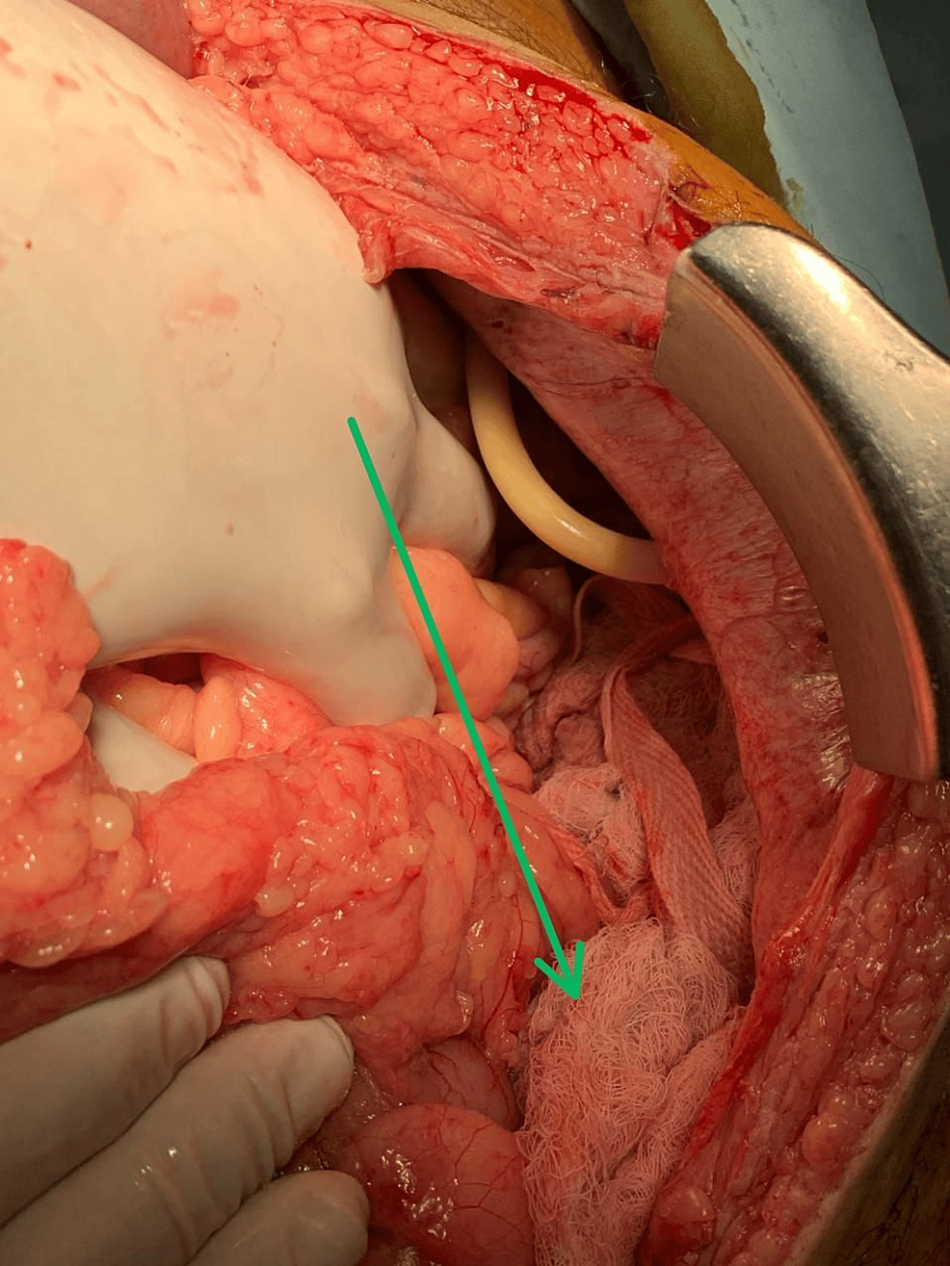
Hepatic packing procedure: managing liver trauma

In cases of severe liver trauma, hepatic packing plays a crucial role in controlling bleeding and stabilising the patient. This surgical intervention involves carefully packing the liver with gauze or other haemostatic materials to apply pressure and staunch the flow of blood from injured vessels. Figure 6 illustrates the meticulous technique and critical nature of hepatic packing in managing traumatic liver injuries.

## Discussion

The preferred imaging modality for diagnosing blunt abdominal traumas is CT. This method's estimated sensitivity is 99%, and its estimated specificity is 96.8% [7]. The therapy of patients with blunt liver trauma has changed, in large part due to the increased availability of helical CT equipment. For the majority of hemodynamically normal patients, conservative treatment can be used with an 80% success rate [8]. Hepatic vein injuries are rare, but they belong to the most dangerous traumas [9]. Thirteen percent of individuals in one of the largest prospective studies of patients with liver traumas had hepatic vein damage. In the study group, the mortality rate for patients with hepatic vein injuries was over twice that of the general mortality rate (61% versus 31%, respectively) [10]. The American Association for the Surgery of Trauma (AAST) categorises liver injuries based on computed tomography (CT) findings. This scale provides a standardised method for classifying the severity of liver trauma, guiding treatment decisions, and making prognostic assessments. Table 2 outlines the AAST's CT Criteria for Liver Injury Scale, offering clinicians a systematic approach to evaluating and managing liver injuries based on imaging characteristics [11].

**Table 2:** The AAST CT criteria for the liver injury scale Adapted from [11]. AAST: American Association for the Surgery of Trauma.

Grade	Criteria
1	Isolated periportal blood tracing, capsular avulsion, superficial parenchymal injury less than 1 cm deep, and subcapsular hematoma less than 1 cm in maximal thickness.
2	1- to 3-cm-deep parenchymal injury and 1- to 3-cm-thick parenchymal/subcapsular hematomas.
3	A parenchymal injury that is deeper than 3 cm and a parenchymal or subcapsular hematoma that is larger than 3 cm.
4	Greater than 10 cm in diameter parenchymal or subcapsular hematoma, lobar damage, or vascular deprivation Grade 5: Complete vascular deprivation or effacement of the liver hepatic avulsions, Grade 6.

Contrast material may extravasate into a parenchymal haematoma locally or freely as a jet into the peritoneal space when there is an active haemorrhage. A significant hepatic venous injury detected on a CT scan should be interpreted as a sign of a serious injury [12]. Liver injury laparotomies are performed in the same way as any other trauma laparotomy. The first line of treatment for liver haemorrhage is typically direct pressure applied using packs. The Pringle manoeuvre, bimanual liver compression, and manual aortic compression above the coeliac trunk are additional approaches. It is essential to use packed cells, platelets, fresh frozen plasma, and cryoprecipitate for intravascular volume replenishment and coagulopathy correction. If necessary, a meaningful examination of the injury should be performed following a Pringle manoeuvre with the application of a vascular clamp, followed by appropriate resuscitation and liver mobilisation [13].

## Conclusions

The case of a 15-year-old female with a Grade 4 hepatic injury and a concurrent Grade 1 renal injury following a 12-foot fall underscores the severity of blunt trauma in adolescents. Prompt recognition and intervention, including emergency laparotomy and hepatic packing, played a crucial role in halting haemorrhage and ensuring successful management. Conservative treatment post-surgery, coupled with magnetic resonance cholangiopancreaticography and confirmation of hepatic parenchymal laceration, facilitated a smooth postoperative course. With vigilant monitoring and adherence to activity restrictions, the patient achieved a favourable outcome, highlighting the importance of comprehensive trauma care in mitigating complications and promoting recovery.
